# Pituitary macroadenoma presenting as severe hyponatremia: a case report

**DOI:** 10.1186/s13256-019-2000-4

**Published:** 2019-02-23

**Authors:** Bopeththe Vidanelage Kameera Madhusanka Bopeththa, S. M. M. Niyaz, Chathuranga Medagedara

**Affiliations:** 1 Emergency Medicine, Teaching Hospital, kandy, Kandy, 70500 Sri Lanka; 2 Department of Surgery, Teaching Hospital, Kandy, Sri Lanka

**Keywords:** Hyponatremia, Hypopituitarism, Pituitary macroadenoma, Syndrome of inappropriate secretion of an antidiuretic hormone

## Abstract

**Background:**

Hyponatremia is defined as a serum sodium level of less than 135 mEq/L in a patient. Although hyponatremia is not an uncommon laboratory finding, especially in the elderly, hunting for the etiology is a challenging issue for any clinician. The three first-line investigations that are required for further analysis are urine osmolality, serum osmolality, and urinary sodium levels in addition to clinical assessment of volume status.

**Case presentation:**

A 69-year-old previously well Sinhalese man presented with lethargy, loss of appetite, vomiting, and altered behavior of 1 week’s duration. An examination revealed Glasgow Coma Scale of 13/15 and marginally low blood pressure. Initial serum sodium level was 104 mmol/L, and plasma and urinary osmolalities were 251 mOsm/kg and 305 mOsm/kg, respectively. His urinary sodium level of 158 mmol/L was suggestive of a clinical picture of a syndrome of inappropriate secretion of antidiuretic hormone. Even after correction of hyponatremia with intravenously administered 3% saline, a persistent altered behavior necessitated cerebral imaging that confirmed the presence of pituitary macroadenoma. Meanwhile, his hormone profile showed very low serum cortisol and low free tetraiodothyronine levels. An ultrasound scan of his abdomen affirmed the presence of normal adrenal glands. With intravenously administered hydrocortisone and orally administered levothyroxine replacement, he showed marked clinical improvement that supported the diagnosis of hypopituitarism.

**Conclusion:**

Hyponatremia in the elderly is not an uncommon presentation. However, etiological diagnosis is a challenging task as there are multiple overlapping differential diagnoses.

## Background

Although hyponatremia is not an uncommon laboratory finding especially in the older population, hunting for the etiology is a challenging issue for a clinician. Pituitary hormone deficiency leading to severe hyponatremia as a result of nonfunctional pituitary macroadenoma is a quite rare presentation in clinical practice. The presence of low serum osmolality, high urinary sodium level, and in the absence of clinical features of hypothyroidism or hypopituitarism, we initially suspected syndrome of inappropriate secretion of antidiuretic hormone (SIADH) in this patient. However, in this case, the presence of persistent neurological symptoms even after correction of hyponatremia prompted us to perform cerebral imaging. If our patient had had normal neurological functions, then we would have proceeded without performing cerebral imaging. Therefore, in the evaluation of hyponatremia, complete biochemical, hormonal, and radiological assessment of the hypothalamic–pituitary–adrenal axis is necessary. Therefore, we considered it worth reporting this case, in order to share this important clinical lesson.

## Case presentation

A 69-year-old previously well Sinhalese man presented with lethargy, loss of appetite, vomiting, and altered behavior that lasted for a week. One week ago, he was apparently well but his family members noticed that he was becoming increasingly lethargic. For an initial few days, they were reluctant to seek medical advice; however, with the onset of new behavioral changes, it was decided to bring him to the hospital. He was a business executive and he had never taken alcohol or smoked tobacco in his life. There was no significant family history of note.

On examination, his body mass index was 19 kg/m^2^. His skin temperature was 37.8 °C. He was confused, with a Glasgow Coma Scale (GCS) of 13/15 and showed evidence of mild dehydration. He had normal skin appearance with normal axillary and pubic hair distribution. His pulse rate was 90 beats per minute and blood pressure was 99/60 mmHg. A cranial nerves examination was normal. Both tone and reflexes of his upper and lower limbs were normal except muscle power of grade 4. Gait assessment was not performed due to low GCS. The rest of the examinations including respiratory and abdomen were unremarkable.

The initial laboratory results were as follows: serum sodium 104 mmol/L, serum potassium 4.3 mmol/L, white cell count 8.8 × 10^9^/L, hemoglobin 9.9 g/dL, platelet count 272 × 10^9^/L, serum creatinine 89 μmol/L, and normal liver function tests. Plasma and urinary osmolalities were 251 mOsm/kg and 305 mOsm/kg respectively. His urinary sodium level was 158 mmol/L. Blood sugar level and serum triglyceride levels were within normal range. Although the duration of symptoms was more than 48 hours, the presence of severe hyponatremia necessitated serum sodium correction with intravenously administered 3% saline. After the first 150 ml bolus of 3% saline, his serum sodium level had risen to 115 mmol/L and there was a slight improvement in his orientation. Since his urine output was satisfactory with stable hemodynamic parameters, normal saline 100 ml/hour was continued.

**Fig. 1:**
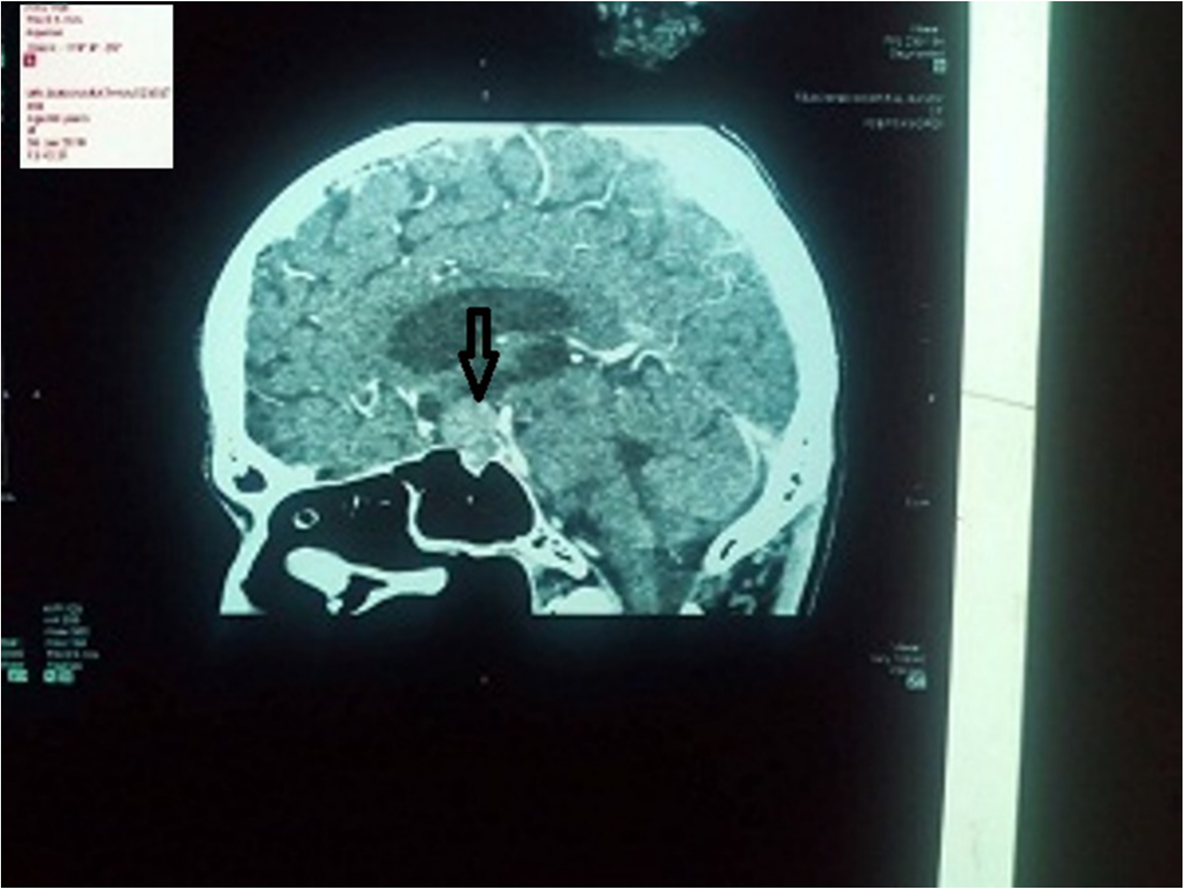
Sagittal section of CT brain showing suprasellar tumor suggestive of pituitary macroadenoma (arrow)

On the second day of admission, his GCS further dropped to 12/15. Repeat serum sodium levels further dropped to 112 mmol/L. Although a rapid correction of serum sodium level is associated with osmotic demyelination syndrome, the presence of severe symptomatic hyponatremia required correction with another 3% saline 150 ml bolus. Repeat serum sodium levels became 120 mmol/L and he showed a slight improvement in GCS of 14/15. Meanwhile, due to fluctuating conscious levels, non-contrast computed tomography (CT) brain was performed. This revealed a mass lesion in the region of optic chiasma and the radiology team suspected an aneurysmal dilation. A CT cerebral angiography was then performed which confirmed the presence of a pituitary macroadenoma (Fig. 1). A pituitary hormone profile was then carried out and the results were as follows: free tetraiodothyronine (T_4_) 8.21 pmol/L (10–68), thyroid-stimulating hormone (TSH) 1.5 mIU/L (0.4–4.6), luteinizing hormone (LH) 1.13 mIU/ml (1.2–7.8), follicular-stimulating hormone (FSH) 1.65 mIU/ml (1.55–9.74), and prolactin 22 ng/ml (3.7–17.9). His morning (9 a.m.) serum cortisol level was 1.49 μg/dL (4.3–22.4). The diagnosis of a nonfunctioning pituitary macroadenoma with secondary hypoadrenalism and hypothyroidism was made. Daily intravenously administered hydrocortisone 50 mg 6 hourly with levothyroxine 75 μg was commenced. After 4 days of replacement, his serum sodium level became stable to around 133 mmol/L and there were marked disappearances of lethargy and fatigability. The intravenously administered hydrocortisone was then replaced with orally administered hydrocortisone and after 1 week of treatment, he had further improved and was able to resume his daily activities as before. He was then referred to the neurosurgical unit for further care. An endoscopic excision of the pituitary tumor was carried out under general anesthesia and later tumor histology revealed pituitary oncocytoma. The preoperative period was covered with intravenously administered hydrocortisone 50 mg 6 hourly and the same dose of levothyroxine.

Following surgery, he was discharged with levothyroxine 75 μg and orally administered hydrocortisone 15 mg two times daily. Six weeks after discharge, his general condition was stable and his serum sodium level was 133 mmol/L. His serum T_4_ level was 34 pmol/L and serum cortisol level was 15 μg/L. Since hormonal levels were well within normal range, the same drug doses were continued. At 3-month clinic and 6-month clinic, visits were unremarkable except the need for reduction in hydrocortisone dose to 10 mg twice daily due to development of impaired blood glucose levels.

## Discussion and conclusion

A 69-year-old previously well man presented with a 1-week history of vomiting, loss of appetite, and confusion. He had severe hyponatremia and his initial biochemical picture suggested a SIADH-like picture. However, even after correction of hyponatremia he had persistent confusion, which necessitated cerebral imaging and proved the presence of a pituitary macroadenoma. With the replacement of hormones, his neurological symptoms disappeared. Later, surgical resection of the tumor was offered and his long-term follow-up was satisfactory with hormone replacement.

Hyponatremia is defined as a serum sodium level of less than 135 mEq/L. Serum sodium below 125 mEq/L is considered severe hyponatremia and is associated with increased morbidity and mortality [1]. According to Mannesse *et al.*, the prevalence of mild and severe hyponatremia in the geriatric population is 22% and 4.5% respectively [2].

An approach to the diagnosis of hyponatremia involves careful history taking and examination including cardiac, pulmonary, renal, endocrine, neurology, malignancy, and drug history, especially diuretics. Also required for further evaluation are: complete serum and urine biochemical tests; pituitary, adrenal, and gonadal hormone assays; and imaging of adrenals and sometimes the pituitary gland. However, with respect to the analysis of etiology of hyponatremia, evaluation of volume status and determination of serum and urine osmolality, including urinary sodium levels, are mandatory [3].

In this patient, hypovolemic hyponatremia was considered because there were dry mucous membranes and marginally low blood pressure. However, the presence of low serum osmolality and high urine osmolality along with high urinary sodium levels led us to consider the high possibility of SIADH, even in the setting of hypovolemic hyponatremia [4]. Primary adrenal insufficiency or hypopituitarism was not considered at the initial evaluation because of the absence of pigmentation, the presence of normal hair distribution, normoglycemia, normokalemia, and ultrasonically normal adrenal glands.

Later, even after correction of hyponatremia, due to persistence of altered behavior, a non-contrast CT scan of our patient’s brain was performed and it showed a mass lesion at optic chiasma, possibly an aneurysmal dilatation. However, CT cerebral angiography confirmed the presence of pituitary macroadenoma. Meanwhile, his serum 9 a.m. cortisol level became available and it was very low. The absence of clinical features of primary adrenal failure together with very low cortisol levels, ultrasonically normal adrenals, and the presence of pituitary macroadenoma, favored the diagnosis of pituitary failure. However, his prolactin level was slightly high, probably due to domperidone or hormone release due to local pressure effects on the pituitary stalk. Finally, with intravenously administered hydrocortisone and levothyroxine replacement, there was a marked resolution of clinical symptoms with stabilization of serum sodium level within the near-normal range, without significant fluctuations. This retrospectively proved our diagnosis.

The clinical presentation of a pituitary tumor can be of hypersecretion of hormones, hyposecretion of hormones, or neurological symptoms. Pituitary tumors with normal pituitary functions usually present like SIADH. Although primary pituitary tumors are associated with hypopituitarism, metastases from adenoid cystic carcinoma of the parotid gland and renal cell carcinomas causing hypopituitarism have also been described in the literature [5].

For the evaluation of pituitary tumors, magnetic resonance imaging (MRI) is the gold standard, and, in our patient, a non-contrast CT scan of his brain showed the details of the tumor. However, some authors have discouraged CT scans of the brain for the evaluation of pituitary tumor [6].

Although some consider hyponatremia a consequence of ageing, it is a serious medical condition associated with increased morbidity and mortality [7]. Under the resolution of clinical symptoms of hyponatremia, even after correction of hyponatremia to reasonable levels, it has to be thoroughly investigated and complete hypothalamic–pituitary–adrenal–gonadal hormone profiles have to be evaluated with the imaging of the pituitary gland.

## Abbreviations

CT: Computed tomography
FSH: Follicular-stimulating hormone
GCS: Glasgow Coma Scale
LH: Luteinizing hormone
MRI: Magnetic resonance imaging
SIADH: Syndrome of inappropriate secretion of antidiuretic hormone
T_4_: Tetraiodothyronine
TSH: Thyroid-stimulating hormone
